# Identification of abnormal protein expressions associated with mouse spermatogenesis induced by cyclophosphamide

**DOI:** 10.1111/jcmm.16263

**Published:** 2021-01-12

**Authors:** Xuexia Liu, Qian Li, Zhixin Wang, Fujun Liu

**Affiliations:** ^1^ Central Laboratory Affiliated Yantai Yuhuangding Hospital of Qingdao University Yantai China; ^2^ Research Department Affiliated Yantai Yuhuangding Hospital of Qingdao University Yantai China

**Keywords:** cyclophosphamide, meiosis, sperm quality, spermatogenesis, testis

## Abstract

Cyclophosphamide (CP) is a clinical anticancer drug that can cause male reproductive abnormalities, but the underlying mechanisms for this remain unknown. The present study aimed to explore the potential toxicity induced by CP in spermatogenesis events of germ cell proliferation, meiosis, and blood‐testis barrier integrity at the molecular level. CP‐treated mice showed significantly reduced serum testosterone levels, sperm motility and concentration. The results of immunohistochemistry and Western blot showed that CP reduced the proliferation of germ cells (PCNA, PLZF) and increased germ cell apoptosis (Bax and TUNEL‐positive cells) in CP‐treated mice testes. The expression of meiotic related proteins (SYCP3, REC8, MLH1) decreased significantly in the fourth week after administration, and the expression of blood‐testis barrier related proteins (β‐catenin, ZO‐1) and sperm quality‐associated proteins (PGK2, HSPA4) decreased significantly in the first week after administration. CP leads to the apoptosis of male germ cells, inhibits the proliferation of germ cells, and affects meiosis and the blood‐testis barrier, resulting in the decline of sperm quality. This study provides information to further the study of molecular mechanism and protective strategy of CP influence.

## 1 INTRODUCTION

Nowadays, male reproductive health is faced with increasing risks from environmental and medical factors.^1^ Paternal exposure to drugs and toxicants may cause aberrant sperm quality, leading to infertility.^2^ Most anticancer drugs commonly used in clinical practice affect male fertility.^3^ Cyclophosphamide (CP) is a widely used chemotherapeutic agent, which produces extensive side effects such as cytotoxicity and infertility.^4^ It may contribute to the impairment of spermatogenesis and sperm quality, thus leading to an increased incidence of azoospermia and oligozoospermia.^5^

Cyclophosphamide disrupts cell growth and differentiation, mainly by cross‐linking DNA strands, and damages the germinal epithelium integrity. It acts on rapidly proliferating tissues, which is its therapeutic basis and toxic properties.^2^ As a known male germ cell toxicant, CP can cause increased DNA damage, oxidative stress, and retention of protamine. Its male reproductive toxicity often manifests as oligospermia, azoospermia, testicular tissue toxicity, alterations in testosterone levels and oxidative stress parameters.^6^, ^7^, ^8^, ^9^, ^10^ As a chemotherapeutic agent, CP‐induced infertility has a profound impact on life quality. Therefore, special attention should be given to maintain fertility following CP‐induced damage. Some protocols, such as hormonal treatment and synthetic and natural antioxidants, have been utilized to protect spermatozoa from CP‐induced toxicity.^11^, ^12^

Although the effects of CP on male fertility are well known, the underlying molecular mechanisms and the biological events mostly affected are poorly understood. High doses of cyclophosphamide (>100 mg/kg BW) can lead to significantly impaired spermatogenesis, including germ cell loss,^4^, ^9^, ^11^ whereas a low dose of cyclophosphamide (6.8 mg/kg BW) has no obvious effects on testicular histology.^13^ Therefore, in order to better understand the effects of cyclophosphamide on spermatogenesis at the molecular level, we used a low dose of cyclophosphamide (10 mg/kg BW) to construct a CP‐treated mouse model and investigated the affected testicular functions and sperm quality. CP‐treated male mice showed poor sperm quality with decreased sperm concentration and progressive sperm motility. These phenotypes may have arisen from impaired testicular functions, such as spermatogonia proliferation, blood‐testis barrier or meiosis. This study provides important information for further understanding of the influence of CP on male reproduction and utilizes an important animal model for male reproduction.

## 2 MATERIALS AND METHODS

### 2.1 Animals

Male Kunming mice (6‐7 weeks old, 30‐32 g, Beijing Vital River Laboratory Animal Technology Co., Ltd.) were housed under the following standard conditions before initiation of the experiment: temperature (23 ± 1°C), humidity (45%‐55%), and photoperiod (12 h/12 h light/dark cycle), and free access to food and water. Animal care and use guidelines for Animal Experimentation were followed. The experiment was approved by the Medical Ethics Committee of Yantai Yuhuangding Hospital (No. 2019‐156).

### 2.2 Construction of CP‐induced mouse model

Mice were randomly classified into two groups consisting of 60 animals each and were treated as follows: group 1: mice were injected intraperitoneally with physiological saline once a day for 5 days; group 2: mice were injected intraperitoneally with cyclophosphamide (determined concentration of 10 mg/kg, bodyweight, C3250000, Sigma‐Aldrich) once a day for 5 days. The injected volume of cyclophosphamide was to be established according to the preliminary experiment and published report.^13^ Six mice were taken for analysis from group 1 and group 2 at 1 day, 1 week, 2 weeks, 4 weeks and 10 weeks after cyclophosphamide administration, respectively. Mice were killed by cervical dislocation. One testis was fixed in formaldehyde (4%) for histological evaluation, and the other was used for protein extraction. The relative weight of the testis was expressed as g/g of bodyweight.

Before euthanasia, serum was collected for the detection of testosterone levels. The serum was stored at −80°C after centrifugation. Serum testosterone was quantified by liquid chromatography‐tandem mass spectrometry.

Spermatozoa from the caudal epididymis were collected. Briefly, caudal epididymides were rapidly removed after euthanasia, cut into small pieces and placed in PBS with 10% (w/v) bovine serum albumin (BSA).The released spermatozoa were collected for analysing sperm parameters.^14^

Histological abnormalities of the testes in CP‐treated mice were examined by haematoxylin and eosin (HE). Paraffin was de‐waxed in three changes of xylene followed by transfer through three changes of 100% ethanol, 90% ethanol and 70% ethanol. After rinsing in running water, the samples were stained in haematoxylin solution followed by eosin solution. The samples were dehydrated and observed under a light microscope (DM LB2, Leica).

### 2.3 Immunohistochemistry (IHC)

Testis slides were processed as described in our previous publication.^14^ Briefly, antigen retrieval was performed by microwaves, and endogenous peroxidase was removed by 3% hydrogen peroxide. After blocking with 3% BSA, the slides were incubated with primary antibodies overnight at 4°C. Primary antibodies were related to proteins of PCNA, PLZF, BAX, PGK2 and HSPA4L (PCNA, ab92552; PLZF, ab189849; BAX, ab32503; PGK2, ab183031; HSPA4L, ab87241, Abcam). After washing with PBS, slides were incubated with horseradish peroxidase (HRP)‐conjugated IgG (Zhong‐Shan Biotechnology) at a final dilution of 1:400 for 1 hour at 37°C. A 3,3′‐diaminobenzidine (DAB) Kit (Zhong Shan Biotechnology) was used to visualize positive staining. Haematoxylin was used to counterstain the sections, and the sections were dehydrated and mounted for examination under bright field microscopy (DM LB2; Leica). Pre‐immune immunoglobulin G (IgG) was used as primary antibody for the negative control. To keep the results consistent, each experiment included CP‐treated sections and control sections at each time‐point, and the results were repeated three times for statistical analysis. Positive immunostaining was used to generate the quantitative results for analysis by ImageJ software. The unequal illumination was processed by shading correction, and a reference slide was used to correct the measurement system. After the immunostaining images were converted to greyscale, average p signal intensity and the relative area of positively stained cells were defined as the average optical density. Ten fields were chosen from each section of each testis, and the average numbers and relative intensities of positive cells were measured for each seminiferous tubule.

### 2.4 TUNEL assay

A one‐step TUNEL apoptosis assay (Beyotime Biotechnology) was used to detect apoptotic cells that undergo extensive DNA degradation during the late stages of apoptosis. Briefly, testicular slides were de‐waxed in xylene, 100% ethanol, 90% ethanol and 70% ethanol, successively. Samples in slides were then reacted with 20 μg/mL Proteinase K (DNase‐free). The samples were washed with PBS and incubated with TUNEL assay solution. Samples were washed with PBS again and observed under Zeiss LSM510 Meta software (LSM5 version 3.2, Carl Zeiss Microimaging).

### 2.5 Western blot

Western blot analysis was done as described previously.^14^ The antibodies used in IHC were also applied to the Western blotting analysis. Primary antibodies were raised against proteins associated with spermatogonia proliferation (PCNA, ab92552; PLZF, ab189849, Abcam), meiosis (SYCP3, ab15093; STRA8, ab49602, Abcam; REC8, D222997; MLH1, D121003; DMC1, D224646, BBI Solutions), the blood‐testis barrier (β‐catenin, ab32572; ZO‐1, ab96587, Abcam), an apoptosis‐associated protein (Bax, ab32503) and sperm quality (PGK2, ab183031; HSPA4L, ab87241, Abcam).

### 2.6 Statistical analysis

All data were analysed using GraphPad Prism 7 (GraphPad Prism). All values were shown as mean ± standard deviation (SD) of three independent experiments. One‐way analysis of variance (ANOVA) was performed to evaluate the means. *P* value < .05 was considered to be statistically significant.

## 3 RESULTS

### 3.1 Construction of CP‐treated mouse model

**FIGURE 1:**
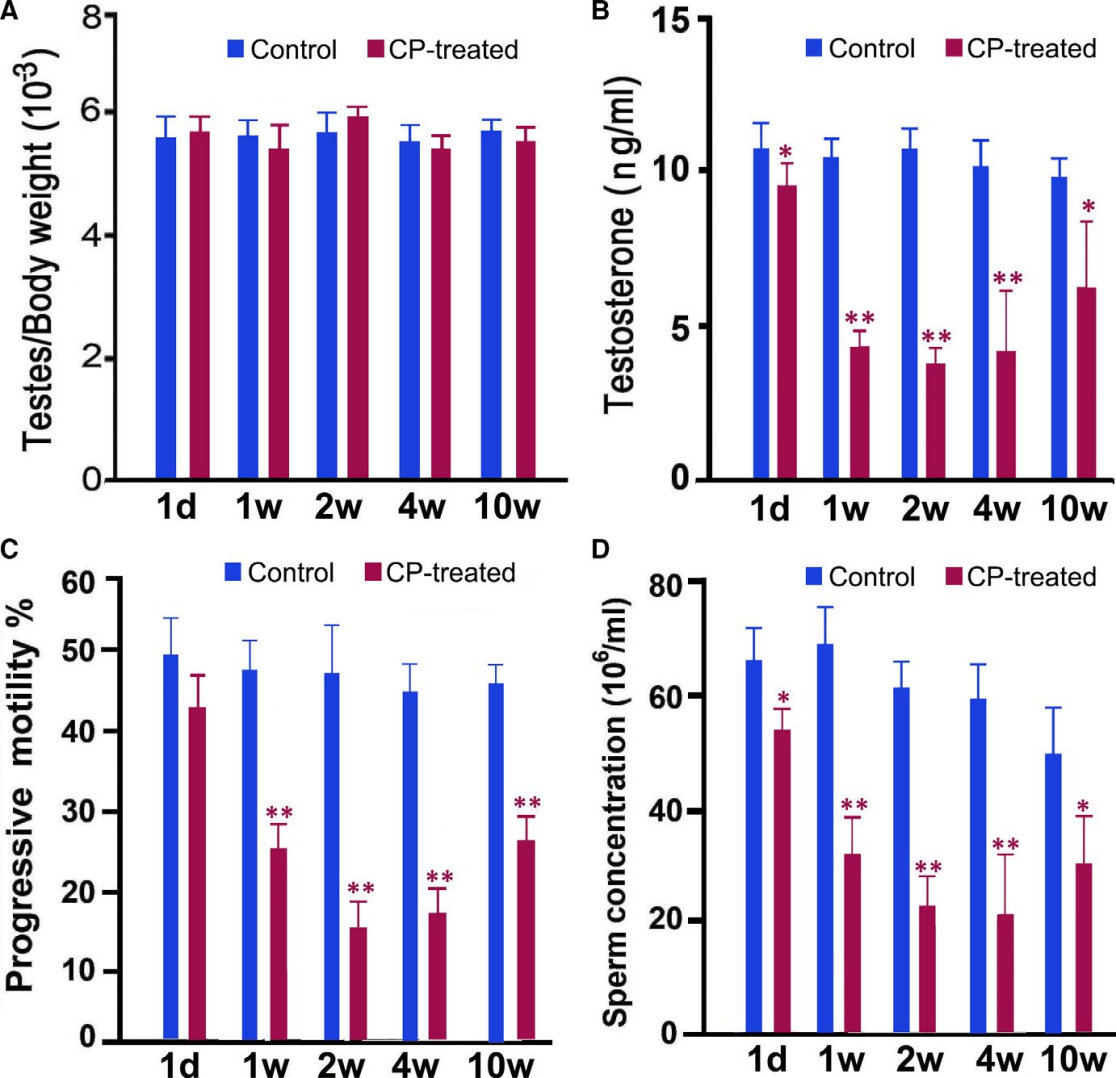
Characteristics of bodyweight (A), testosterone concentration (B), and sperm motility (C) and concentration (D) in CP‐treated mice at different time‐points after CP treatment. For each time‐point after CP treatment, CP‐treated mice were compared with saline‐treated control mice. Data of each group were obtained from six mice and analysed by one‐way ANOVA; *P < .05, **P < .01

**FIGURE 2:**
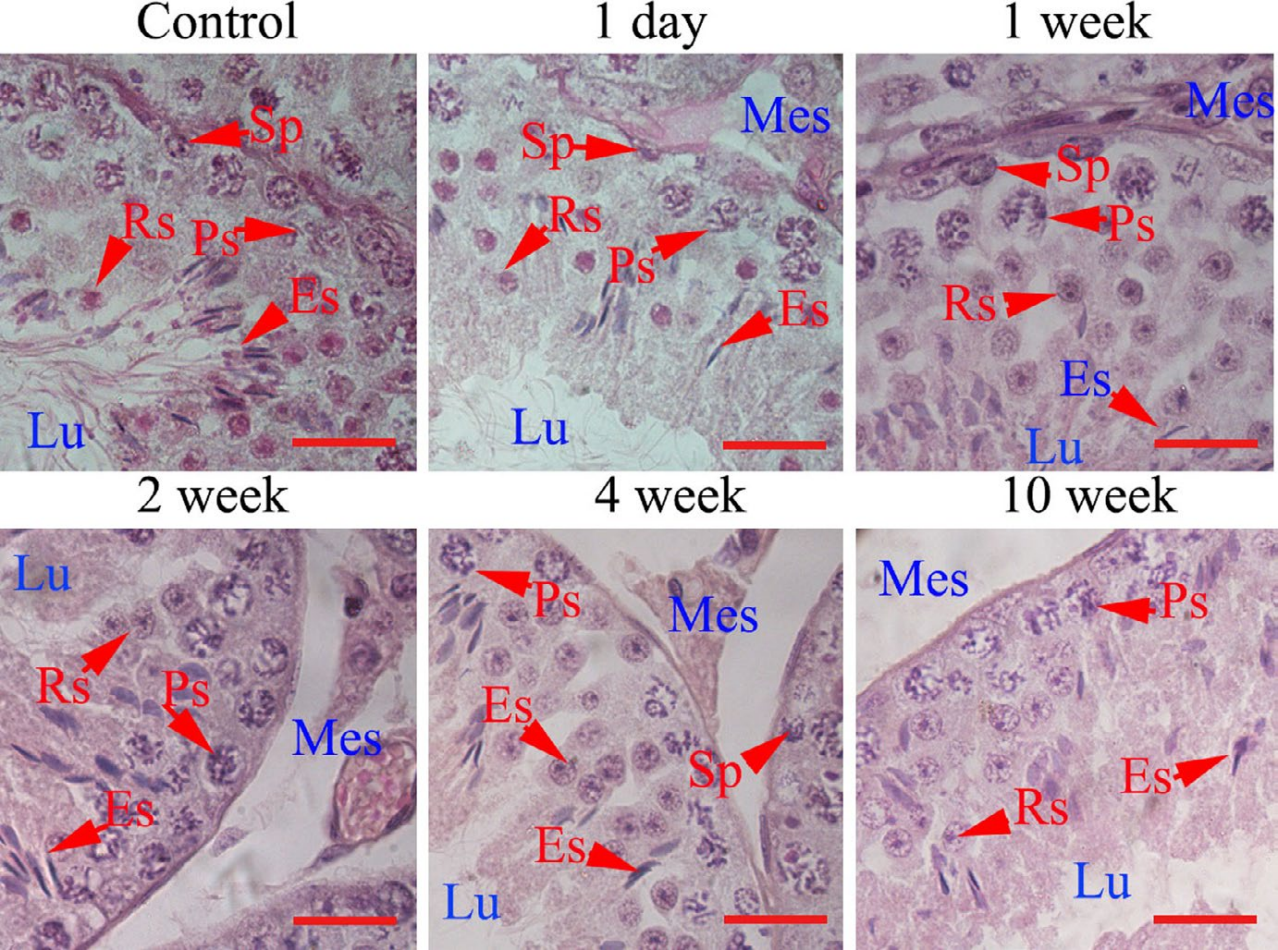
Morphological analysis of mice testes. Testes were obtained from control and CP‐treated mice at different time‐points after CP treatment and stained with haematoxylin‐eosin. The representative control image was obtained from saline‐treated mice at 1 d. Es, elongated spermatid; Lu, tubule lumen; Mes, mesenchyme; Ps, pachytene spermatocyte; Rs, round spermatid; Sp, spermatogonia. Each bar represents 10 μm

A CP‐treated mouse model was constructed by intraperitoneal injection of CP for five consecutive days. We selected five time‐points (1 day, 1 week, 2 weeks, 4 weeks and 10 weeks) after CP treatment to study the effects of CP on spermatogenesis and sperm quality. A general characteristic analysis demonstrated that no obvious changes in ratio of testes to bodyweight were observed among the different groups (Figure 1A); however, serum testosterone, sperm motility and sperm counts showed a decreasing trend from 1 day after CP treatment. Particularly, significantly decreased values were observed from 1 to 4 weeks after CP treatment (Figure 1B,C,D). Morphological analysis showed loose mesenchymal structure and decreased numbers of germ cells in seminiferous tubules from 2 weeks post‐injection (Figure 2).

### 3.2 Effects of CP on germ cell proliferation

**FIGURE 3:**
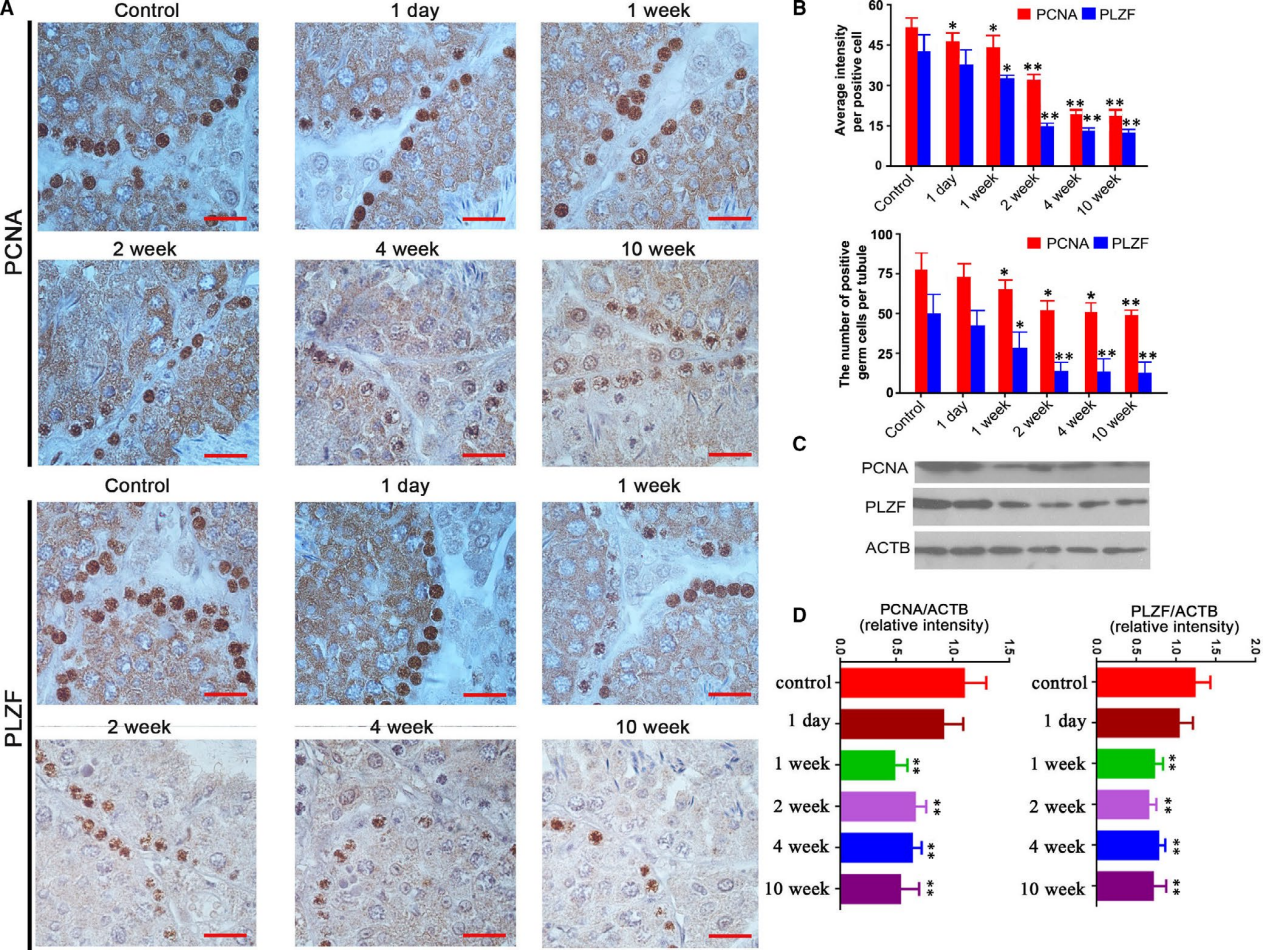
Expressions of PCNA and PLZF in testes from the control and CP‐treated mice at different time‐points after CP treatment. A, Representative images of cellular localization PCNA and PLZF in the control and CP‐treated mice testes, the control image was from saline‐treated mice at 1 d; B, the numbers of PCNA and PLZF‐positive germ cells, and their average staining intensities in positive germ cells; C, Western blot analysis of PCNA and PLZF in the control and CP‐treated mice testes; and D, Quantification of PCNA and PLZF expressions; the control was loaded by the pooled sample from saline‐treated mice. Quantification of blotting intensity is shown as a ratio of the grey value of each band to the control. Quantitative evaluation was performed by ImageJ and analysed by one‐way ANOVA; *P < .05, **P < .01. Each bar represents 10 μm

Protein markers associated with spermatogonia and spermatocyte proliferation (PCNA, PLZF) were examined in CP‐treated mice testes. Compared to the control mice, PCNA and PLZF‐positive germ cells had irregular arrangements in CP‐treated mice (Figure 3A). The significantly reduced PCNA‐positive spermatogonial and spermatocyte cells and PLZF‐positive spermatogonial cells were observed at 1 week after CP treatment. In additional, the average intensity of PCNA‐positive germ cells significantly decreased from 1 day after CP treatment, and significant decrease of PLZF‐positive cells was observed from 1 week after CP treatment (Figure 3B). Western blot analysis also showed the significant decreased expressions of PCNA and PLZF in CP‐treated mice testes from 1 week on after CP treatment (Figure 3C,D).

### 3.3 TUNEL assay and expression of Bax

**FIGURE 4:**
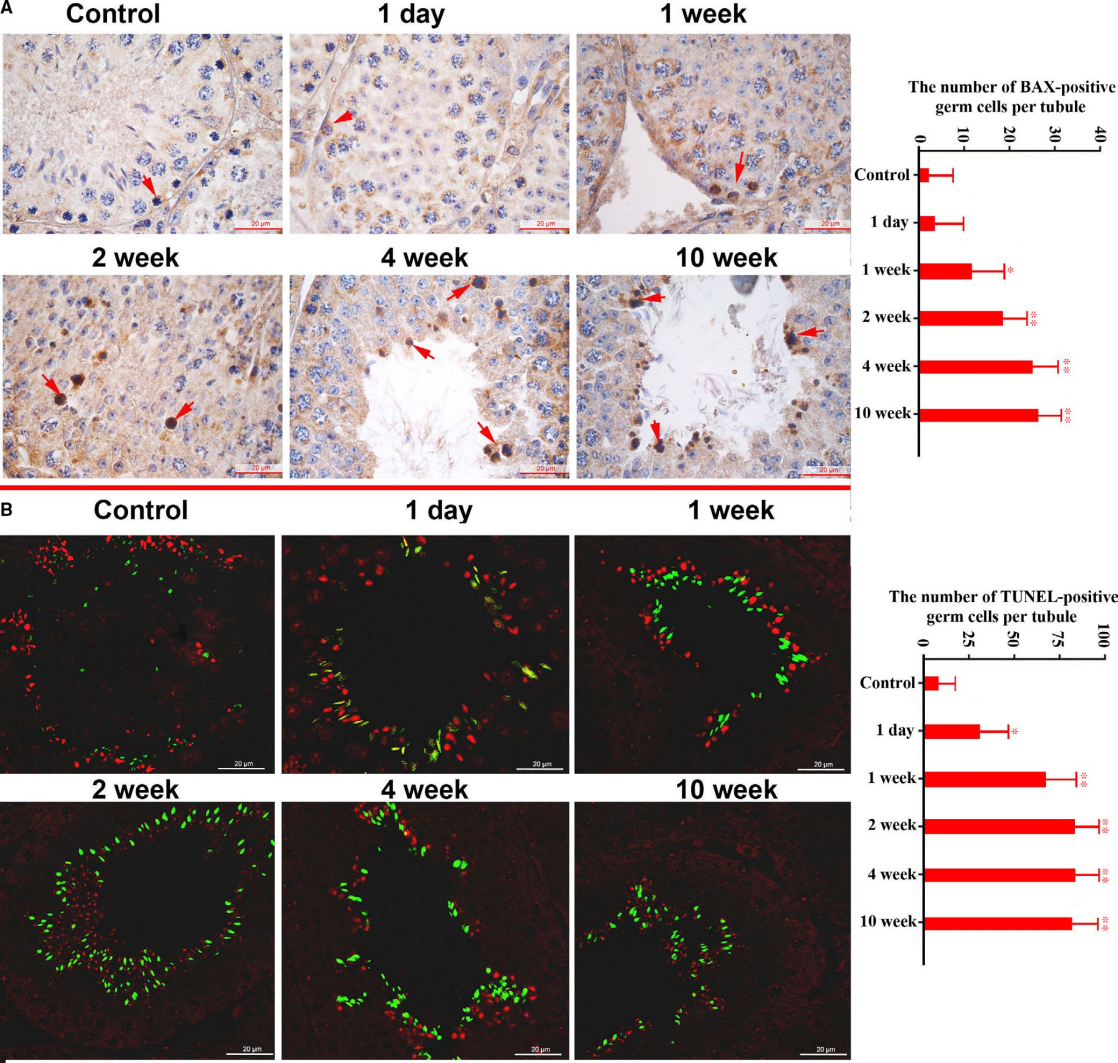
Immunohistochemical localization of Bax (A) and TUNEL detection (B) of germ cell apoptosis in the control and CP‐treated mice at different time‐points after CP treatment. A, Representative staining images of Bax‐positive germ cells (arrows indicated Bax‐positive cells) and the average number of positive cells per tubule. B, Representative staining images of TUNEL analysis and the average number of positive cells per tubule. The red image shows the nucleus stained by PI, and the green ones are the apoptotic cells. Control values were obtained from averages of control mice at five time‐points. The statistical analysis was performed by one‐way ANOVA; *P < .05, **P < .01. Each bar represents 20 μm

TUNEL assay using immunofluorescence and BAX expression detection using immunohistochemistry were performed to evaluate apoptotic germ cell in CP‐treated testes. Compared with the control, no significant alterations in staining intensities of TUNEL‐positive or BAX‐positive germ cells were observed in CP‐treated mice testes. However, significantly increased numbers of BAX‐positive germ cells, including spermatogonia, spermatocyte and elongated spermatid, were observed in CP‐treated mice testes from 1 week after CP injection, and increasing TUNEL‐positive germ cells were observed from 1 day after CP injection. These apoptotic cells could also be detected up to 10 weeks after drug withdrawal (Figure 4).

### 3.4 Expressions of meiosis‐associated proteins in CP‐treated mice testes

**FIGURE 5:**
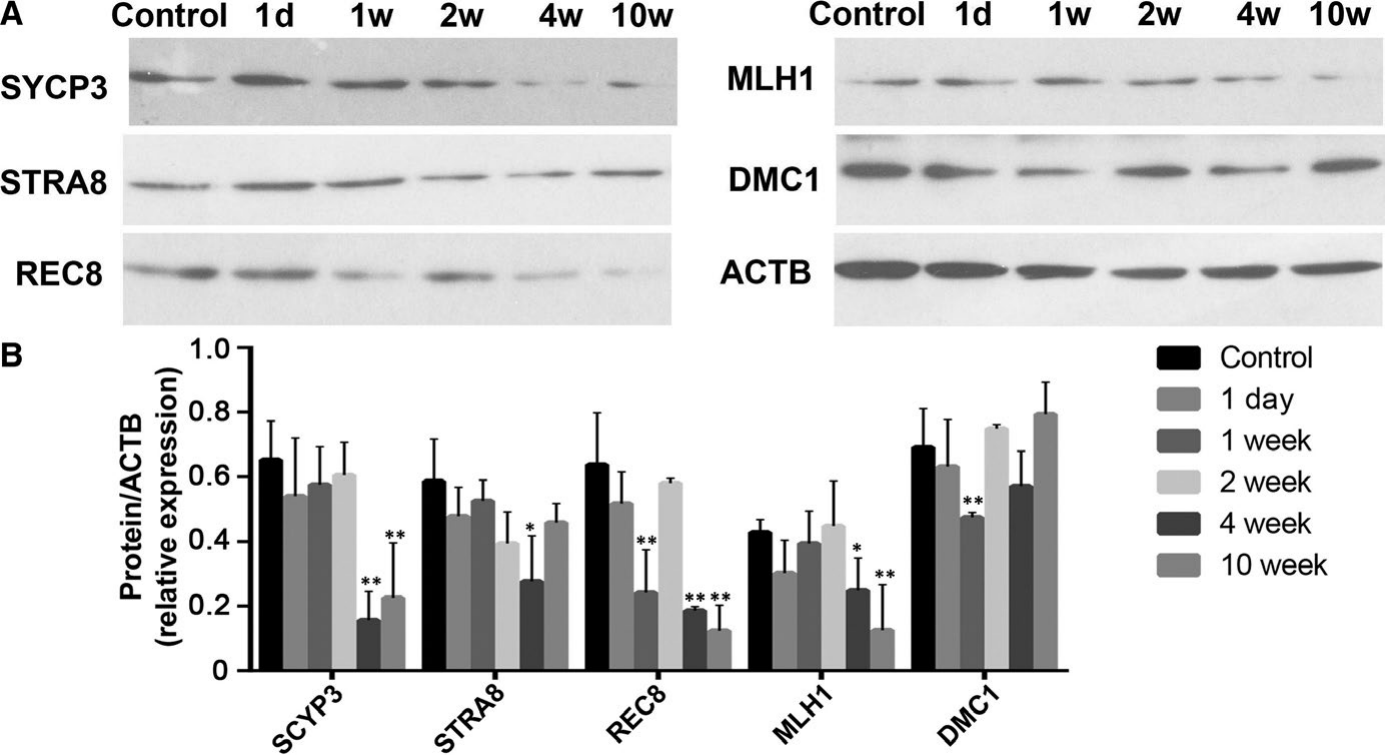
Western blot analysis of meiosis‐associated proteins in testes from the control and CP‐treated mice at different time‐points after CP treatment. A, Representative image of Western blotting; B, quantification of band intensity. The control was loaded by the pooled saline‐treated mice; quantification of blotting intensity is shown as a ratio of the grey value of each band to the control. Quantitative evaluation was performed by ImageJ and compared by one‐way ANOVA; *P < .05, **P < .01

Expressions of key markers associated with meiosis (SYCP3, STRA8, REC8, MLH1, DMC1) were detected by Western blot. As shown in Figure 5, these proteins showed altered expressions in mice testes after CP treatment. Among them, SYCP3, REC8 and MLH1 were significantly down‐regulated in testes at 4 and 10 weeks after CP treatment (Figure 5).

### 3.5 Effects of CP on blood‐testis barrier

**FIGURE 6:**
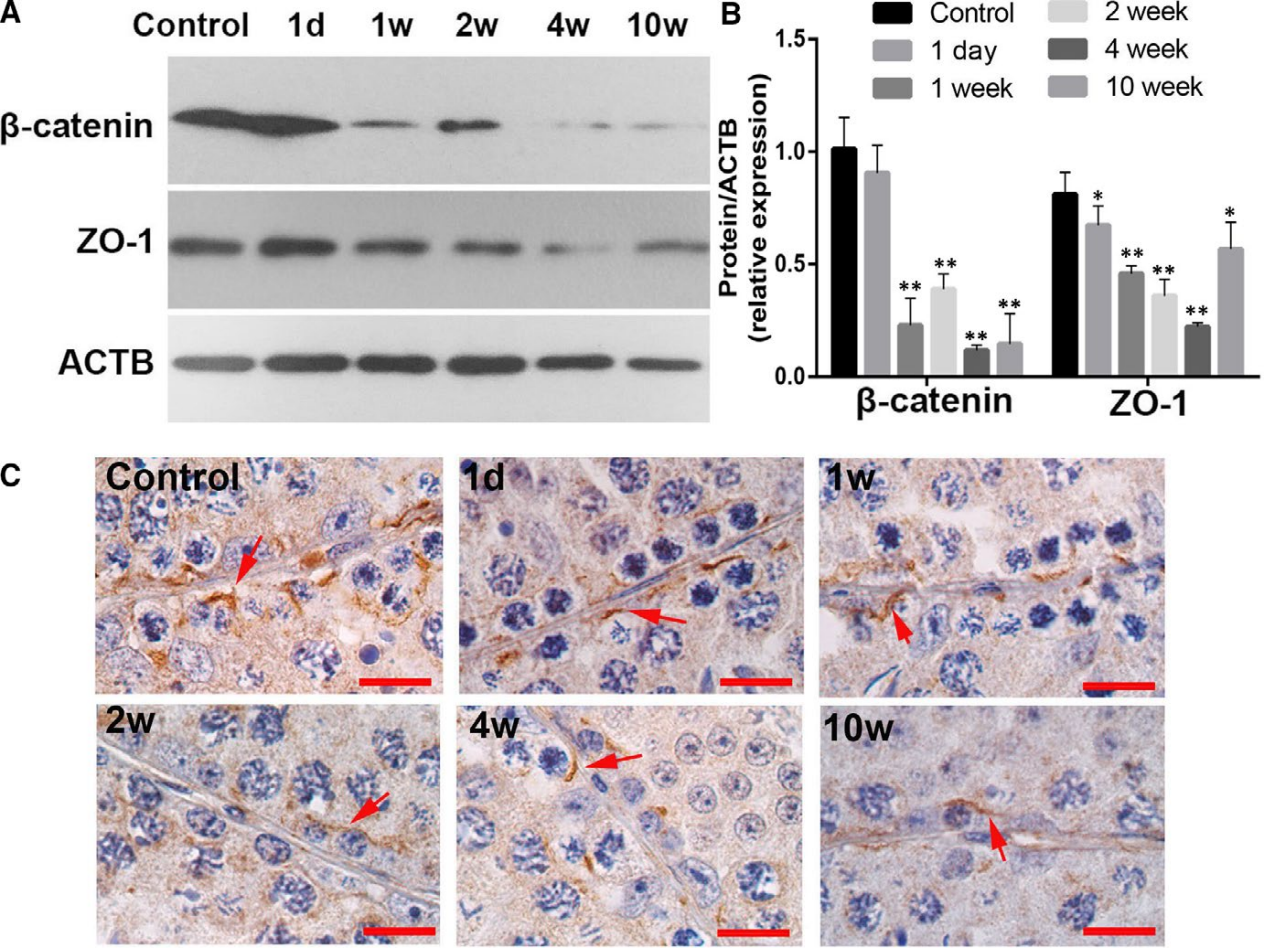
Expressions of β‐catenin and ZO‐1 in testes from the control and CP‐treated mice at different time‐points after CP treatment. A, Representative image of Western blotting; B, quantification of band intensity. The control was loaded by the pooled saline‐treated mice. The control was loaded by the pooled saline‐treated mice. Quantification of blotting intensity is shown as a ratio of the grey value of each band to the control; and C, representative cellular localization of β‐catenin in CP‐treated mice testes. The arrows show the positive cellular localization of β‐catenin in CP‐treated mice testes, and each bar represents 10 μm. Quantitative evaluation was performed by ImageJ and compared by one‐way ANOVA; *P < .05, **P < .01

ZO‐1 and β‐catenin are biomarkers for blood‐testis barrier maintenance. As shown in Figure 6, β‐catenin and ZO‐1 showed the decreased expression levels after CP treatment. β‐catenin was mainly expressed in the cell surface of Sertoli cells and was significantly down‐regulated in CP‐treated mice testes from 1 to 10 weeks. Meanwhile, ZO‐1 showed significant down‐regulated expression from 1 week after CP treatment.

### 3.6 Expressions of PGK2 and HSPA4L in CP‐treated mice testes

**FIGURE 7:**
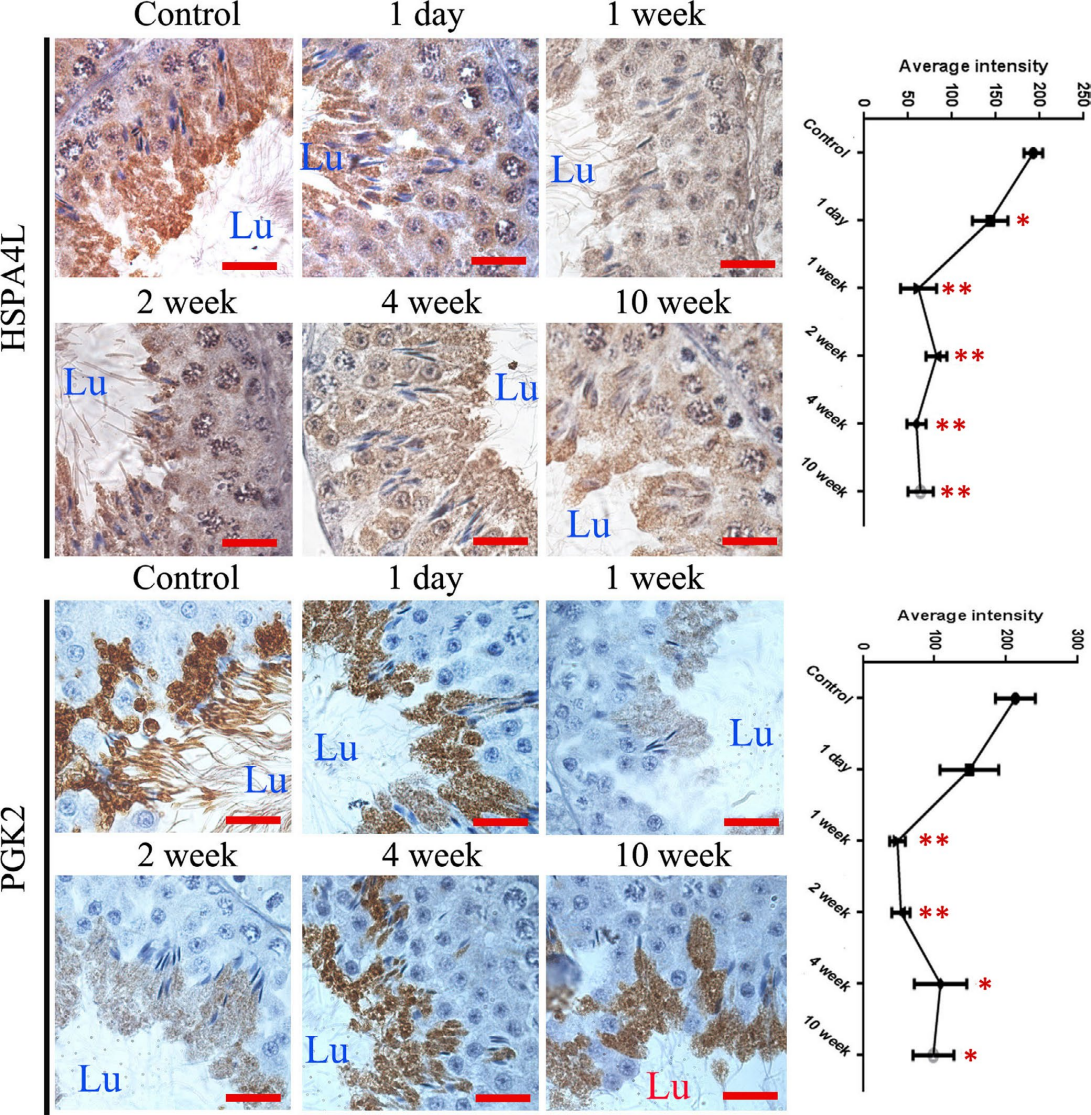
Immunohistochemical analysis of HSPA4L and PGK2 in testes from control and CP‐treated mice at different time‐points after CP treatment. A, Representative images of cellular localization of HSPA4L and PGK2 in the testes; B, quantitative intensity of HSPA4L and PGK2 in the testes. Quantitative evaluation was performed by ImageJ and compared by one‐way ANOVA; *P < .05, **P < .01. Lu, tubule lumen. Each bar represents 10 μm

PGK2 and HSPA4L are testis‐specific proteins, which are associated with sperm quality. Their expressions in CP‐treated mouse testes were examined by immunohistochemistry. As shown in Figure 7, PGK2 and HSPA4L were mainly expressed in round and elongated spermatids, and their expressions were significantly down‐regulated in CP‐treated testes from 1 week post‐injection.

## 4 DISCUSSION

Despite clear evidence of sub‐fertility induced by CP in male mice, there is no detailed understanding of its underlying effects on spermatogenesis. A clear characteristic of CP‐induced biological events in spermatogenesis would provide useful information to elucidate the effects of CP on male fertility and effectively use the CP‐treated mouse model to perform male reproductive research.

In the present study, we constructed a CP‐treated mouse model through continuous administration of a low dose of CP for 5 days, then detected the general phenotype and key spermatogenesis events from 1 day to 10 weeks after drug withdrawal. Spermatogenesis determines the quality of matured sperm and further affects male fertility.^15^ The main effects of spermatogenesis on sperm quality are sperm motility and sperm concentration.^16^ CP‐treated mice had poor sperm quality with decreased sperm progressive motility and sperm counts, which make them a suitable mouse model for the study of asthenospermia. This model can be used to investigate the underlying mechanisms of some key molecules participating in the regulation of sperm quality. No significant alterations to relative testis weights were observed in CP‐treated mice, but significantly decreased serum testosterone levels were observed CP‐treated, which may lead to impaired spermatogenesis processes.^17^ Testosterone, one of the most important steroid hormones produced by testicular Leydig cells, is vital for the development of the spermatogenesis and is actively involved in key spermatogenesis processes, such as germ cell proliferation, meiosis and the blood‐testis barrier.^18^ It can also promote sperm production in the testis. Until 10 weeks after CP withdrawal, serum testosterone, sperm motility and concentration did not return to normal levels. These results indicate that CP has long‐term and chronic effects on spermatogenesis.

Morphological analysis indicated that testicular structures of CP‐treated mice were affected from 2 weeks after injection, which may be the result of decreased serum testosterone from 1 day on after CP withdrawal. In the present study, we detected the cell proliferation markers PCNA and PLZF, which are well‐known biomarkers associated with spermatogonia and spermatocytes proliferation.^19^, ^20^ Decreased numbers of PCNA and PLZF‐positive germ cells and expression levels suggest that the proliferation of spermatogonia and spermatocytes were mainly affected, which could explain the decrease in sperm concentration and implicate the affection of subsequent meiosis. Bax, which is involved in germ cell apoptosis, is up‐regulated when testis suffer from damage stress.^21^, ^22^ Results showed that CP caused a significant increase in TUNEL‐positive cell numbers of round and elongated spermatids, as well as Bax‐positive cells in spermatogonia, spermatocyte and elongated spermatids, which may regulate germ cell apoptosis and reflect the toxic effects of CP.

The effects of CP on spermatogonia and spermatocyte proliferation will further affect the meiosis activity. A panel of established biomarkers for meiosis were detected in CP‐treated mice testes. Of them, STRA8 is an essential initiator of meiosis and is used as a marker for differentiating spermatogonia and preleptotene spermatocytes,^23^ whereas SYCP3 is a marker for meiotic spermatocytes.^24^ RCE8 is regarded as the main complex required for centromeric cohesion.^25^ MLH1 is well known to be associated with meiosis,^26^ and DMC1 is a recombinase that is essential for meiotic synapsis.^27^ It has been estimated that faulty meiosis occurs in 5%‐25% of human germ cells, which develop into aneuploid gametes, and is thought to be the reason for at least 35% of pregnancy losses and mental retardation cases.^28^ In CP‐treated mice, SYCP3 and REC8 were significantly down‐regulated from 4 weeks onward after CP withdrawal. As shown in our previous study and the results of reduced PCNA‐positive germ cell in CP‐treated testes,^29^ spermatogonia and spermatocytes may be severely affected, leading to subsequent reduction in meiosis and associated protein expressions. This suggests that CP administration may also affect the key meiosis process, which may contribute to sperm quality problems such as decreased sperm concentration.

The blood‐testis barrier (BTB) creates a specialized microenvironment necessary for germ cell development and movement,^30^ especially depending on its intercellular endothelial tight junctions (TJs). Damage to the BTB causes germ cell loss, reduced sperm count, male infertility or sub‐fertility.^31^ β‐catenin and ZO‐1 are well‐known protein markers for BTB integrity.^32^, ^33^ Down‐regulated expressions of β‐catenin and ZO‐1 in CP‐treated mice testes imply the impaired function of BTB caused by CP treatment.

The altered expressions of marker molecules of cell proliferation, meiosis and the blood‐testis barrier indicate damages to the key spermatogenesis processes, consequently affecting sperm production and sperm quality. Previously, we identified PGK2 and HSPA4L as key spermatogenesis proteins that were related to sperm quality.^14^, ^34^ Here, significantly down‐regulated expression of PGK2 and HSPA4L was also observed in CP‐treated mice testes, which implied poor spermatogenesis and sperm quality in CP‐treated mice.

In conclusion, administration of CP to adult male mice lowers serum testosterone concentration and sperm quality. This study identified altered protein markers associated with key spermatogenesis processes following CP exposure, which reflects the influence of CP on spermatogenesis and sperm quality and also provides marker molecules for the study of the molecular mechanism of CP and strategies to protect against its damaging effects.

## CONFLICT OF INTEREST

The authors declare that there is no conflict of interest.

## AUTHOR CONTRIBUTION

**Xuexia Liu:** Data curation (equal); Investigation (equal); Methodology (equal); Writing‐original draft (equal); Writing‐review & editing (equal). **Qian Li:** Data curation (equal); Investigation (equal); Methodology (equal). **Zhixin Wang:** Data curation (equal); Investigation (equal); Methodology (equal). **Fujun Liu:** Conceptualization (equal); Data curation (equal); Investigation (equal); Supervision (equal); Writing‐original draft (equal); Writing‐review & editing (equal).

## Data Availability

Data sharing is not applicable to this article as no new data were created or analysed in this study.
